# Supplementary material for: Chromothripsis during telomere crisis is independent of NHEJ, and consistent with a replicative origin
Source: Genome Res. 2019 May;29(5):737–49. doi: 10.1101/gr.240705.118 (PMC6499312; doi:10.1101/gr.240705.118)
Supplement: Supplemental Material [file supp_gr.240705.118_Supplemental_file_1.zip › contigs/annotated_contigs/DB114/contig.2.DB114_length_285_mean_cov_3.0.docx]

**DB114_length_285_mean_cov_3.0**

```
TTACCCTCTTCCAGAATGAGATCTGGGCCAGCGGTGGATGCCGCCCCTCCAAGGAGAGAGGAAGCATCTCGCTGGTAGCTGGAACTTAG
 >chr11:85477523-85477706 - E=8e-69
GCAAGCCTCTTGGTGCTTCCCAAGCTGTGTAGTTAATAAAAGCCCATGGATATGGTGGCTTTTGCAGCAGCTTCATGCAATATCAGAAC

TCTGT|ACTG|CATCCAGAATCACGTTGAGGAGGCCGTCTTTGAAGTATAATGTCTGCTTGGTGCACCACAGGTACTCCTCCTCTGTTT
          >chr11:85568418-85568516 - E=1e-47
CGCCCTTCCAGGCGTACAAC
```
